# Future Risk After Achieving Clinical Remission in Patients with Severe Asthma: A Comparison of Different Definitions

**DOI:** 10.3390/jcm14228201

**Published:** 2025-11-19

**Authors:** Mami Rikimaru, Junpei Saito, Yasuhito Suzuki, Masami Kikuchi, Suguru Sato, Atsuro Fukuhara, Ryuki Yamada, Yoko Shibata

**Affiliations:** Department of Pulmonary Medicine, Fukushima Medical University School of Medicine, Fukushima 960-1295, Japan

**Keywords:** severe asthma, biologics, clinical remission, exacerbation, future risk

## Abstract

**Background**: Clinical remission (CR) has recently been recognized as an important treatment goal in severe asthma. However, there is no consensus on its definition. **Objectives**: We aimed to evaluate differences in the rates of CR based on various definitions and to investigate the extent to which exacerbations, lung function decline, and sustained CR are observed over 12 months after evaluating CR, to identify the best definition reflecting future risks. **Methods**: Severe asthmatic patients receiving biologic therapy (*n* = 52) were retrospectively evaluated for 24 months according to three previously reported CR definitions incorporating oral corticosteroid (OCS) use, exacerbations, symptoms, and lung function. **Results**: Over 24 months, significant improvements were observed in all clinical parameters. At 12 months, the CR rates according to the three definitions ranged from 26.9% to 40.4%. At 24 months, sustained CR was achieved in 17.6% to 29.4%. Two of the three definitions (CR (no OCS use, no exacerbations, and well-controlled asthma (ACT ≥ 23) and CR (no OCS use, no exacerbations, ACT ≥ 20, and ΔFEV_1_ ≥ 100 mL from baseline)) predicted a reduced risk of future exacerbations. Annual FEV_1_ decline did not differ between CR and non-CR groups, regardless of the three definitions. **Conclusions**: Defining CR based on three components—no OCS use, no exacerbations, and well-controlled asthma (ACT ≥ 23)—may provide prognostic information for predicting future exacerbation risk in severe asthmatic patients receiving biologic therapy.

## 1. Introduction

It is estimated that there are more than 250 million patients with asthma worldwide, of whom approximately 5 to 10% have severe refractory asthma. In Japan, a study using a health insurance claims database reported that 7.8% of Japanese asthma patients were classified as having severe asthma [1], which is consistent with global data [2,3,4]. Of these, 2.5% had uncontrolled severe asthma. These patients represent a key population with significant unmet needs for asthma care, and effective management strategies are required to achieve better disease control and quality of life, leading to reduce both the clinical and economic burden.

Since the introduction of omalizumab in 2003, five biologic agents have been introduced as novel treatments for asthma in Japan, leading to remarkable improvements in the management of severe asthma. The clinical effectiveness of these biologics has been extensively evaluated in numerous randomized clinical trials, demonstrating significant reductions in annual exacerbations, decreased oral corticosteroid (OCS) use, improvements in symptoms and lung function, along with favorable long-term clinical outcomes and safety profiles [5,6]. These positive results have led to a paradigm shift in asthma management strategies toward a more ambitious multidimensional treatment goal: the potential achievement of clinical remission (CR) of asthma [7]. However, there are currently no standardized, globally acceptable criteria for CR in asthma. Several national guidelines regarding asthma include proposed criteria for achieving CR; ≥12 months with no exacerbations, no OCS use, no clinically significant symptoms, and stable and optimized lung function [8,9,10]. More recently, some studies have defined CR using two approaches—with and without a lung function criterion [11,12]. One plausible explanation for this variability in CR definitions is that there is no consensus on the duration and components of sustained long-term asthma control, nor on how to assess future risks such as frequent exacerbations and annual declines in lung function once CR is achieved.

In this study, we pursued two main objectives: (1) to compare the rates of CR attained using various definitions, and (2) to determine which definition of CR best reflects long-term asthma control based on two key outcomes assessed during the 12 months following the initial achievement of CR—the occurrence of exacerbations and lung function decline.

## 2. Materials and Methods

### 2.1. Study Design

This was a retrospective, longitudinal, observational study conducted at the outpatient clinic of Fukushima Medical University Hospital. Patients with severe asthma consecutively undergoing add-on biologic therapy between May 2009 and January 2023 were enrolled in the study. Demographic and clinical data were collected at baseline (T0) and at 6 months (T6), 12 months (T12), and 24 months (T24) after the initiation of biologic therapy. The study was approved by the Research Ethics Committee of Fukushima Medical University (Approval No. REC2024-203, approved on 13 May 2025) and was conducted in accordance with the Declaration of Helsinki. As this study was retrospective and non-invasive, informed consent was obtained from patients through an opt-out approach: information about the study was made publicly available via the university’s official website, allowing patients the opportunity to opt out of participation.

### 2.2. Patient Population

Patients over 18 years of age who met the diagnostic criteria for severe asthma defined by the American Thoracic Society/European Respiratory Society (ATS/ERS) were enrolled in the study [13]. All patients had been receiving high-dose inhaled corticosteroids (ICS) and long-acting β2-adrenergic agonists (LABA), often in combination with a long-acting muscarinic receptor antagonist (LAMA), a leukotriene receptor antagonist (LTRA), sustained release theophylline, or OCS. The use of other biologics in treatment prior to study entry was not considered as part of the inclusion criteria.

### 2.3. Data Collection

Demographic and clinical data were collected from electronic medical records at T0, T6, T12, and T24 after initiation of biologic therapy. Clinical data included the number of exacerbations, pharmacological therapies, Asthma Control Test (ACT) scores [14], pulmonary function test results, fractional exhaled nitric oxide (FeNO) levels, and white blood cell counts with differentials.

Pulmonary function tests (Chestac-11 Cyber S-type; Chest M.I., Inc., Tokyo, Japan) were performed based on the ATS/ERS guidelines [15]. Data on postbronchodilator forced expiratory volume in one second (FEV_1_, FEV_1_% predicted), forced vital capacity (FVC), and FEV_1_/FVC ratio were retrieved. FeNO measurements were performed according to the Japanese Respiratory Society recommendations [16] and the ATS/ERS recommendations before pulmonary function tests [17].

### 2.4. Definition of Asthma Exacerbation

Patients with moderate or severe acute exacerbations were classified as having asthma exacerbation according to the ATS/ERS statement [18]. Severe exacerbations were defined as requiring treatment with oral prednisolone, an increase in the maintenance dose of prednisolone for ≥3 days, hospitalization, or an emergency department visit because of asthma. Moderate exacerbations were defined as having one or more of the following for ≥2 days consecutively: deterioration in symptoms, deterioration in peak expiratory flow (PEF) levels, or increased use of rescue bronchodilators that was not severe enough to warrant systemic corticosteroid use.

### 2.5. Definitions of Clinical Remission

In this study, we applied the following three previously reported definitions of clinical remission (CR) and examined the clinical parameters included in each definition at four assessment points: T0, T6, T12, and T24 after the initiation of biologic therapy.

♦ Four-component definitions
  - CR_(ACT≥20+ΔFEV1≥100)_: no annual exacerbations + no OCS use + ACT ≥ 20 + ΔFEV_1_ ≥ 100 mL from baseline [19]
  - CR_(ACT≥20+%FEV1≥80)_: no annual exacerbations + no OCS use + ACT ≥ 20 + FEV_1_ ≥ 80% predicted [20]
♦ Three-component definition
  - CR_(ACT≥23)_: no annual exacerbations + no OCS use + ACT ≥ 23 [21]

Furthermore, sustained CR, defined as meeting the above CR criteria at T12 and maintaining this status through T24 was also assessed.

### 2.6. Statistical Analysis

Continuous variables were expressed as the median and interquartile range (IQR) or the mean ± standard deviation, and categorical variables were expressed as numbers (n) and percentages (%). The normality of data distribution was checked using the Shapiro–Wilk test and the Kolmogorov–Smirnov test. Comparisons of clinical parameters from baseline to 24 months were performed using the Wilcoxon signed-rank test and the Friedman test. The overall study population was divided into two groups according to whether sustained on-treatment CR was achieved at T12. Comparisons of continuous variables between CR and non-CR group were performed using the Mann–Whitney *U* test or the Kruskal–Wallis test with Bonferroni correction when appropriate. Fisher’s exact test or the chi-square test was used to compare categorical variables. Receiver operating characteristic (ROC) curves were constructed to compare the three CR definitions for predicting the future risk of exacerbations, between T12 and T24. Statistical significance was set at *p* < 0.05. Statistical analyses were performed using SPSS for Windows (version 27.0; IBM, Armonk, NY, USA) and GraphPad Prism (version 5.0 for Windows; GraphPad Software, San Diego, CA, USA).

## 3. Results

### 3.1. Characteristics of Enrolled Patients

Fifty-two patients with severe asthma who met the inclusion criteria were enrolled. Of these, 18 patients received omalizumab, seven received mepolizumab, 10 received benralizumab, and 17 received dupilumab. Table 1 shows the baseline clinical characteristics of the enrolled patients. Overall, 53.8% of the patients were female, the median age was 63 years, and 65.4% were never-smokers. Asthma control in this population was generally poor, with a median of 3.0 (IQR 1.0–5.0) exacerbations per year, a median predicted FEV_1_ of 67.5% (IQR 49.7–80.9), and a median ACT score of 17 (IQR 14–20). Regarding type 2 inflammatory biomarkers, the median blood eosinophil count was 436 /µL, sputum eosinophils 48%, serum IgE 367 IU/mL, and FeNO 71 ppb, suggesting that the most patients had type 2 inflammation despite adequate treatment for severe asthma. Approximately 70% of patients had allergic rhinitis, and 40% had chronic rhinosinusitis. All patients were treated with high-dose ICS plus LABA, either alone or in combination with additional anti-asthmatic medications such as LAMA or LTRA. In addition, 36.5% of patients received maintenance doses of prednisolone, and 28.9% had previously received other biologics prior to enrollment.

### 3.2. Changes in Clinical Parameters for Assessing CR from Baseline to T24

To assess the efficacy of biologic therapies, changes in exacerbation frequency, FEV_1_, ACT scores, and OCS use were examined over 24 months.

**Figure 1:**
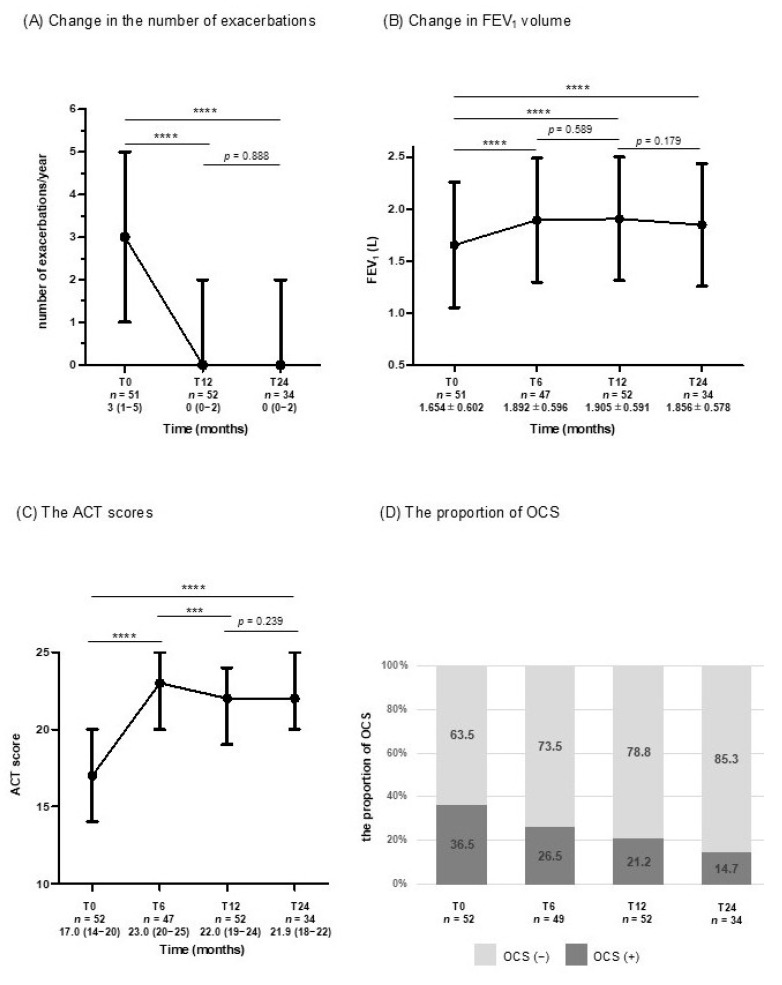
Changes in clinical parameters from baseline to 24 months: (**A**) number of exacerbations per year (median and IQR); (**B**) FEV_1_ (L) (mean ± SD); (**C**) ACT score (median and IQR); (**D**) proportion of patients using OCS (%). (*** *p* < 0.001; **** *p* < 0.0001). Abbreviations: T0 = baseline; T6 = 6 months; T12 = 12 months; T24 = 24 months; IQR, interquartile range; FEV_1_, forced expiratory volume in one second; SD, standard deviation; ACT, Asthma Control Test; OCS, oral corticosteroid.

The number of exacerbations showed a rapid and significant reduction to 0 (IQR 0–2) during the first year (*p* < 0.0001), and this reduction was maintained over the following year (Figure 1A).

FEV_1_ levels also significantly improved from a baseline value of 1.65 ± 0.60 L to 1.89 ± 0.60 L at T6 (*p* < 0.0001), and sustained up to T24 (Figure 1B).

ACT scores showed a significant improvement from 17.0 (IQR 14–20) at baseline to 23.0 (IQR 20–25) at T6 (*p* < 0.0001), exceeding the threshold for well-controlled asthma (ACT ≥ 20). This favorable control was maintained, with scores of 22.0 (IQR 19–24) at T12 (*p* = 0.001) and 21.9 (IQR 18–22) at T24 (*p* = 0.239) (Figure 1C).

The proportion of patients using OCS significantly decreased from 36.5% at T0 to 14.7% at T24 (Figure 1D).

Notably, 50 out of the 52 patients showed significant improvement in at least one of the four indices mentioned above.

### 3.3. Proportion of Patients Achieving CR at T12

**Figure 2:**
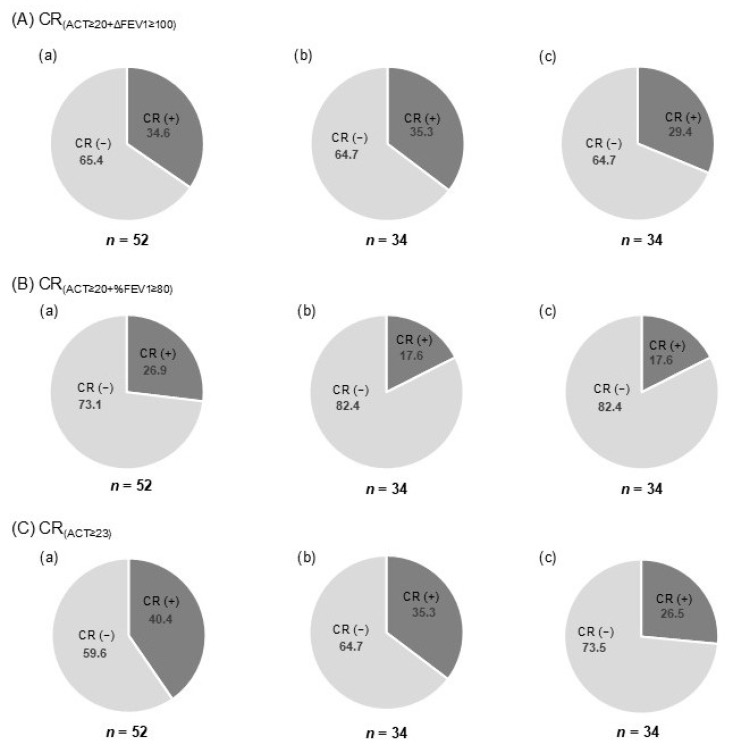
Proportion of patients achieving clinical remission (CR) based on three CR definitions: (**a**) at 12 months, (**b**) at 24 months, and (**c**) with sustained remission during months 12–24. Abbreviations: OCS, oral corticosteroid; ACT, Asthma Control Test; FEV_1_, forced expiratory volume in one second; CR, clinical remission; CR_(ACT≥20+ΔFEV1≥100)_, No annual exacerbations + no OCS use + ACT ≥ 20 + ΔFEV_1_ +100 mL from baseline; CR_(ACT≥20+%FEV1≥80)_, No annual exacerbations + no OCS use + ACT ≥ 20 + FEV_1_% predicted ≥ 80%; CR_(ACT≥23)_, No annual exacerbations + no OCS use + ACT ≥ 23.

Figure 2 shows the proportion of patients who achieved the CR according to the three definitions at 12 months after initiation of biologic therapy. In all enrolled patients, the rates of CR_(ACT≥20+ΔFEV1≥100)_ and CR_(ACT≥20+%FEV1≥80)_ were 34.6% and 26.9%, respectively (Figure 2(Aa,Ba)), while the rate of CR_(ACT≥23)_ was 40.4% (Figure 2(Ca)), suggesting that the three-component definition tended to yield higher CR rates than the four-component definitions.

**Figure 3:**
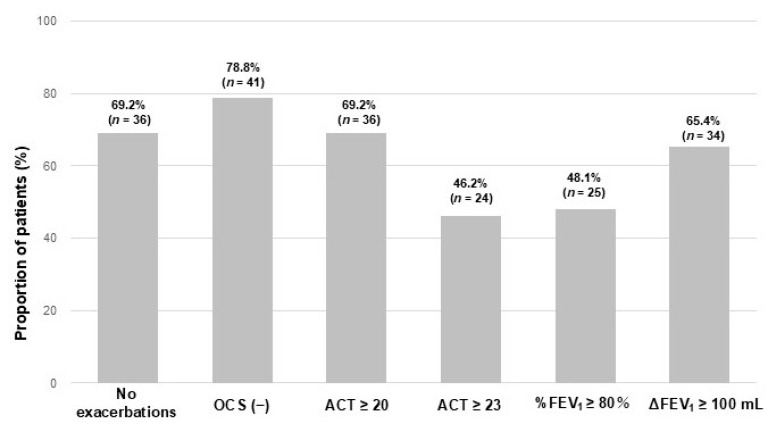
Proportion of patients showing improvement in each component of CR definitions. Abbreviations: OCS, oral corticosteroid; ACT, Asthma Control Test; FEV_1_, forced expiratory volume in one second; %FEV_1_, FEV_1_% predicted; ΔFEV_1_ ≥ 100, FEV_1_ ≥ 100 mL from baseline.

Among the individual components of the CR criteria, failure to achieve an ACT score ≥23 (46.2%) and FEV_1_% predicted ≥80% (48.1%) were the predominant factors associated with non-attainment of CR (Figure 3).

### 3.4. Proportion of CR and Sustained CR at T24 According to Each Definition

At T24, the rates of CR according to the three definitions—CR_(ACT≥20+ΔFEV1≥100)_, CR_(ACT≥20+%FEV1≥80)_, and CR_(ACT≥23)_—were 35.3%, 17.6%, and 35.3%, respectively (Figure 2(Ab,Bb,Cb)). These rates were comparable to or slightly lower than those at T12. Moreover, the sustained CR rates at T24 in the same patients were 29.4%, 17.6%, and 26.5%, respectively (Figure 2(Ac,Bc,Cc)), which were lower than the corresponding CR rates at T12 and T24.

### 3.5. Comparison of CR Definitions for Predicting Exacerbations Between T12 and T24 After Achievement of CR at T12

**Figure 4:**
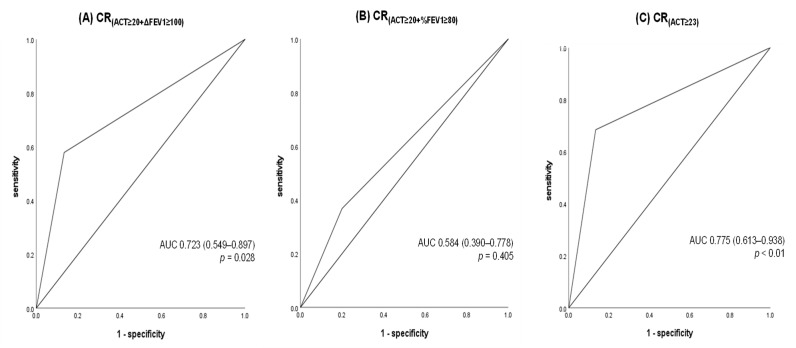
Receiver operating characteristic (ROC) curves for predicting the future risk of exacerbations and proportion of patients who experienced asthma exacerbations during mouths 12–24 according to each CR definition. Abbreviations: ROC, Receiver Operating Characteristic; CR, clinical remission; ACT, Asthma Control Test; FEV1, forced expiratory volume in one second; %FEV_1_, FEV_1_% predicted; ΔFEV_1_ ≥ 100; FEV_1_ ≥ 100 mL from baseline; OR, odds ratio; AUC, area under the curve.

Patients who achieved CR at T12 under the definitions of CR_(ACT≥20+ΔFEV1≥100)_ and CR_(ACT≥23)_ experienced significantly fewer exacerbations over the following 24 months than those who did not (*p* = 0.013 and *p* = 0.002, respectively). On the other hand, no significant difference in exacerbation frequency was observed between patients who met the definition of CR_(ACT≥20+%FEV1≥80)_ and those who did not (Table 2). Furthermore, there was no significant difference in the predictive accuracy for exacerbations during T12–T24 between CR_(ACT≥20+ΔFEV1>100)_ and CR_(ACT≥23)_ (*p* = 0.558) (Figure 4).

### 3.6. Annual FEV_1_ Change Between T12 and T24 in CR-Positive and CR-Negative Patients at T12

**Figure 5:**
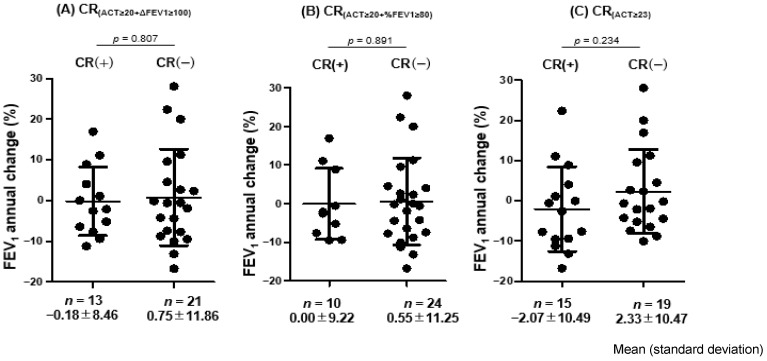
Changes in FEV_1_ between months 12–24 according to CR status at 12 months. Abbreviations: CR, clinical remission; ACT, Asthma Control Test; FEV_1_, forced expiratory volume in one second; %FEV_1_, FEV_1_% predicted; ΔFEV_1_ ≥ 100; FEV_1_ ≥ 100 mL from baseline.

There were no significant differences in the change in FEV_1_ from T12 to T24 between the CR-positive and CR-negative patients at T12, regardless of the CR definition applied (Figure 5).

## 4. Discussion

This is a retrospective observational study which investigated the long-term CR rate over a 24-month period following the initiation of biologic therapy in patients with severe asthma, based on the three previously reported definitions of CR. Over the first 12 months, the patients experienced significantly fewer exacerbations, reduced OCS use, and notable improvements in both FEV_1_ and asthma control. Furthermore, these improvements were largely maintained up to 24 months. At T12, the CR rate for the three-component definition—CR_(ACT≥23)_—was higher compared to the four-component definitions—CR_(ACT≥20+ΔFEV1≥100)_ and CR_(ACT≥20+%FEV1≥80)_—AT T24, the CR rates were 35.3%, 17.6%, and 35.3%, respectively. However, the sustained CR rates in the same patients declined to 29.4%, 17.6%, and 26.5%, respectively, indicating the challenge of maintaining the long-term efficacy of biologic therapy. Among the three CR definitions, CR_(ACT≥20+ΔFEV1≥100mL)_ and CR_(ACT≥23)_ were more predictive of the absence of exacerbations over 24 months.

There are currently no international standards for evaluating the efficacy of biologic therapy in asthma. In previous clinical trials and real-world studies, biologic efficacy has typically been evaluated using one or more outcome measures, including exacerbation frequency, pulmonary function, OCS dosage, asthma symptoms, and quality of life (QOL). Additionally, the Global Evaluation of Treatment Effectiveness (GETE) has also been used as a physician-assessed measure of response [22]. Recent evidence indicates an overall response rate is approximately 60–80% [23]. However, even among patients who no longer experience exacerbations, a considerable number still have low FEV_1_, require maintenance OCS therapy, or present with persistent asthma symptoms. Therefore, whether biologic therapy should be considered effective and continued in such cases remains debatable. Furthermore, both the Global Initiative for Asthma (GINA) guideline [24] and Japanese asthma management guidelines published from Japanese Society of Allergology [25] emphasize not only the absence of symptoms and exacerbations but also the importance of reducing future risks, such as maintaining stable lung function. In other words, until recently, attention in asthma management has been directed mainly toward the short-term efficacy of biologic therapy. Against this background, and with the increasing number of patients with severe asthma receiving biologic therapies, attention has been shifting toward evaluating the long-term sustainability of treatment efficacy. In this context, the concept of “clinical remission” has emerged as a potential treatment goal for biologic therapy, as suggested by Menzies-Gow et al. [26]. Consequently, the national guidelines of several countries have introduced their own definitions of CR, and CR rates based on these definitions have been increasingly evaluated and reported [27].

In the present study, 50 of 52 patients (96.2%) showed improvement in at least one component of the three CR definitions—namely, no exacerbations, good asthma control, stable FEV_1_, and no OCS use—within 12 months following the initiation of biologic therapy. Overall, these components demonstrated significant improvement from baseline to T12, and the favorable effects were sustained at T24. Therefore, when each component was evaluated individually, they appeared to be useful indicators for assessing short-term treatment response. This finding is consistent with the recommendation of the current GINA and Japanese asthma guidelines [24,25], which suggest evaluating the effectiveness of biologic therapy within approximately four months after initiation, in order to determine whether to continue, discontinue, or switch the biologic treatment. We also evaluated CR rates using definitions consisting of three or four components, as proposed in the national guidelines of Germany, Italy, and Japan. The T12 CR rates according to the two four-component definitions were 34.6% and 26.9%, which were comparable to previously reported rates, including 32.1% by Milger K et al. [19] and 20.3% by Perez-de-Llano et al. [28]. Similarly, the T12 CR rate of 40.4% defined by the three-component definition was consistent with previously reported rates, which ranged from 37.6 to 44.9%, depending on the ACT or ACQ cut-off values used in each study [19,29,30]. These previous reports, along with the present findings in the study, suggest that the definitions of CR including pulmonary function criteria result in lower CR rates compared to those that do not incorporate pulmonary function [28,30]. Notably, among patients with baseline FEV_1_% predicted <80%, only 28.9% (11 out of 38) achieved FEV_1_% predicted ≥80% at T12. This low rate of normalized FEV_1_ may reflect the presence of airway remodeling, which is often observed in patients with severe asthma requiring biologics. Taken together, these findings, including ours, indicate that CR rates vary widely depending on the definition used and the characteristics of the study population [31]. Therefore, a major unresolved issue is the lack of data clarifying which aspects of CR, as reflected in the components of its various definitions, are associated with long-term outcomes, including sustained CR in subsequent years and reduction in future risks.

In our study, the CR rates at T24 were 35.3% based on the four-component definition [CR_(ACT≥20+ΔFEV1≥100)_], 17.6% based on another four-component definition [CR_(ACT≥20,%FEV1≥80)_], and 35.3% based on the three-component definition [CR_(ACT≥23)_]. These rates remained similar to those observed at T12, except for [CR_(ACT≥20,%FEV1≥80)_], which showed a lower CR rate. However, sustained CR rates in the same patients were lower across all three definitions: 29.4%, 17.6%, and 26.5%, respectively. Only a few studies have evaluated long-term and sustained CR rates. In these studies, CR rates at T12 and T24 ranged from 30 to 37.2% and 42.8 to 45%, respectively. Sustained CR rates from T12 to T24 ranged from 14.6% to 42.1%. These findings suggest that long-term CR and sustained CR rates vary depending on the follow-up duration, CR definitions, and biologic agents used [30,32,33,34], which are consistent with our findings. Nevertheless, they have not clarified which criteria are most strongly associated with sustained remission or future asthma risks such as exacerbations and annual decline in FEV_1_. To address this gap, in the present study, we investigated whether achieving CR at T12, based on the three definitions, could predict long-term asthma control outcomes, specifically including sustained CR over the subsequent 12 months after T12. Our results demonstrated that one of the four-component definitions [CR_(ACT≥20+ΔFEV1≥100)_] and the three-component definition [CR_(ACT≥23)_] equally predicted the absence of exacerbations in the subsequent year. This suggests that these definitions may serve as practical and clinically meaningful assessment tools for long-term asthma control. In addition, our findings underscore the utility of a symptom-based approach (ACT ≥ 23) as a practical indicator of sustained asthma control. This may have meaningful implications for real-world clinical practice, especially in primary care settings where pulmonary function testing is not available. Therefore, regular evaluation of patient-reported symptoms could guide and help maintain long-term disease control, even in resource-limited settings. On the other hand, in the present study, none of the CR definitions were able to predict annual decline in FEV_1_, one of the major future risks in asthma. This may be explained by the heterogeneity of airflow limitation in patients with severe asthma; some have reversible obstruction, whereas others exhibit fixed airflow limitation due to airway remodeling. Further validation through large-scale, multicenter prospective studies will be essential to confirm the generalizability and clinical utility of our findings.

This study has several limitations. First, it was a single-center, retrospective observational study in which CR rates were evaluated across multiple biologic therapies; as a result, the sample size for each biologic was relatively small. Although future investigations focusing on individual biologics are warranted, our findings may serve as a valuable pilot study to guide the design of larger multicenter studies [29,33,34,35]. Second, tezepelumab was not included in the analysis because it had not been approved in Japan during the observation period with its long-term effects on CR remaining unevaluated. Third, this study did not compare CR outcomes with those in patients with severe asthma who were not receiving biologic therapy. In addition, if biologic therapy was switched before the study entry, carry-over effects may have influenced the results. However, there were no significant differences in CR rates among the three definitions between patients who newly initiated biologics and those who switched from other biologics. Therefore, the influence of carry-over effects is considered to be limited. Further research is needed to determine whether the current definitions of CR are applicable across varying disease severities and treatment contexts, including in patients with mild to moderate asthma and those not treated with biologics.

## 5. Conclusions

Evaluating CR based solely on three components—no OCS use, no exacerbations, and well-controlled asthma (ACT ≥ 23), may provide potentially useful prognostic information on predicting future exacerbation risk in patients with severe asthma requiring biologic therapy. Further large-scale, prospective multicenter studies are warranted to clarify our findings.

## Figures and Tables

**Table 1:** Patient demographic and clinical characteristics before biologic therapy initiation.

	Total Population (*n* = 52)
Age (years)	63 (55–69)
Sex (male/female), *n* (%)	24 (46.2)/28 (53.8)
BMI (kg/m^2^)	23.3 (21.5–29.6)
Duration of asthma (years)	15 (9–21)
Smoking history (never/former/current), *n* (%)	34 (65.4)/16 (30.8)/2 (3.8)
Exacerbations in the past year	3.0 (1.0–5.0)
Blood eosinophils (/μL)	436.0 (125.3–748.5)
Blood IgE (IU/mL)	367.0 (153.0–708.0)
Sputum eosinophils (%)	48.0 (11.0–71.7)
Sputum neutrophils (%)	31.4 (6.1–49.0)
FeNO (ppb)	71.0 (36.9–126.1)
FEV_1_ (L)	1.53 (1.21–1.98)
FEV_1_/FVC ratio (%)	60.8 (50.5–71.6)
FEV_1_% predicted	67.5 (49.7–80.9)
ACT score (points)	17 (14–20)
Initial treatments	
- ICS/LABA, *n* (%)	52 (100)
- ICS dose (fluticasone equivalent), µg/day	1000 (640–1400)
- LAMA, *n* (%)	37 (71.2)
- LTRA, *n* (%)	43 (82.7)
- Omalizumab, *n* (%)	18 (34.6)
- Mepolizumab or Benralizumab, *n* (%)	17 (32.7)
- Dupilumab, *n* (%)	17 (32.7)
- Xanthine derivatives, *n* (%)	34 (65.4)
- Maintenance OCS therapy, *n* (%)	19 (36.5)
- OCS daily dose, mg/day	0.0 (0.0–5.0)
Previous biologics	
- Omalizumab, *n* (%)	8 (15.4)
- Mepolizumab or Benralizumab, *n* (%)	7 (13.5)
- Dupilumab, *n* (%)	0 (0.0)
Comorbidities	
- Childhood asthma, *n* (%)	5 (9.6)
- Allergic rhinitis, *n* (%)	35 (67.3)
- Chronic rhinosinusitis, *n* (%)	22 (42.3)
- Atopic dermatitis, *n* (%)	3 (5.8)
- Allergic conjunctivitis, *n* (%)	8 (15.4)
- Anxiety/depression, *n* (%)	4 (7.7)
- NERD, *n* (%)	3 (5.8)

Data are presented as *n* (%), mean (SD), or median (range). Abbreviations: BMI, body mass index; IgE, total immunoglobulin E; FeNO, fractional exhaled nitric oxide; ppb, parts per billion; FEV_1_, forced expiratory volume in one second; FVC, forced vital capacity; ACT, Asthma Control Test; ICS, inhaled corticosteroid; LABA, long-acting β-agonist; LAMA, long-acting muscarinic antagonist; LTRA, leukotriene receptor antagonist; OCS, oral corticosteroid; NERD, non-steroidal anti-inflammatory drugs exacerbated respiratory disease.

**Table 2:** Number of patients with asthma exacerbations between T12 and T24 according to clinical remission status at T12.

(A) no annual exacerbations + no OCS use + ACT ≥ 20 + ΔFEV_1_ ≥ 100 mL from baseline			
	Exacerbations (+)	Exacerbations (−)	Total
Clinical remission at 12 months (+)	2	11	13
Clinical remission at 12 months (−)	13	8	21
Total	15	19	34
	OR = 0.112, *p* = 0.013		
(B) no annual exacerbations + no OCS use + ACT ≥ 20 + FEV_1_% predicted ≥ 80%			
	Exacerbations (+)	Exacerbations (−)	Total
Clinical remission at 12 months (+)	3	7	10
Clinical remission at 12 months (−)	12	12	24
Total	15	19	34
	*p* = 0.451		
(C) no annual exacerbations + no OCS use + ACT ≥ 23			
	Exacerbations (+)	Exacerbations (−)	Total
Clinical remission at 12 months (+)	2	13	15
Clinical remission at 12 months (−)	13	6	19
Total	15	19	34
	OR = 0.071, *p* = 0.002		

## Data Availability

All data supporting the findings of this study are contained within the article.

## Abbreviations

The following abbreviations are used in this manuscript:

ACQ	Asthma Control Questionnaire
ACT	Asthma Control Test
ATS	American Thoracic Society
AUC	Area Under the Curve
BMI	body mass index
CI	confidence interval
CR	clinical remission
ERS	European Respiratory Society
FeNO	Fractional exhaled nitric oxide
FEV_1_	Forced expiratory volume in 1 s
FVC	Forced vital capacity
GETE	Global Evaluation of Treatment Effectiveness
GINA	Global Initiative for Asthma
ICS	inhaled corticosteroid
IgE	total immunoglobulin E
IQR	Interquartile range
JRS	Japanese Respiratory Society
LABA	long-acting β-agonist
LAMA	long-acting muscarinic antagonist
LTRA	leukotriene receptor antagonist
NERD	non-steroidal anti-inflammatory drugs exacerbated respiratory disease
OCS	oral corticosteroid
OR	odds ratio
PEF	peak expiratory flow
ppb	parts per billion
QOL	quality of life
ROC	receiver operating characteristic
SD	standard deviation
T0	baseline
T12	12 months
T24	24 months
%FEV_1_	FEV_1_% predicted
